# Recurrent Positive Reverse Transcriptase–Polymerase Chain Reaction Results for Coronavirus Disease 2019 in Patients Discharged From a Hospital in China

**DOI:** 10.1001/jamanetworkopen.2020.10475

**Published:** 2020-05-28

**Authors:** Rujun Hu, Zhixia Jiang, Huiming Gao, Di Huang, Deyu Jiang, Fang Chen, Jin Li

**Affiliations:** 1 Department of Emergency, Affiliated Hospital of Zunyi Medical University, Zunyi, China; 2 Department of Nursing, Affiliated Hospital of Zunyi Medical University, Zunyi, China

## Abstract

This case series examines patients with coronavirus disease 2019 (COVID-19) who were discharged from the hospital and had recurrent positive reverse transcriptase–polymerase chain reaction (RT-PCR) results.

## Introduction

Coronavirus disease 2019 (COVID-19) has become pandemic. Previous studies^1,2,3^ on COVID-19 have been mainly centered on the epidemiology, clinical characteristics, radiological features, and treatment of patients with confirmed infection. Follow-up studies of discharged patients have been rarely reported.

## Methods

This case series study was approved by the institutional review board of the Affiliated Hospital of Zunyi Medical University. The need for informed consent was waived because the data were entirely anonymized and the individuals could not be identified. This study follows the Strengthening the Reporting of Observational Studies in Epidemiology (STROBE) reporting guideline.

We collected the clinical data of patients who had been cured and discharged from a hospital designated for patients with COVID-19 in Guizhou Province, China, between January 25, 2020, and February 26, 2020. All COVID-19 infections were classified into 4 different types—mild, moderate, severe, and critical—on the basis of the disease severity.^4^ Patients could be discharged if they met discharge standards.^4^ They were required to quarantine for 14 days in a designated hospital,^4^ and their nasopharyngeal swabs were usually collected on the 7th and 14th days; however, swabs were collected anytime if the patients had clinical symptoms. Real-time reverse transcriptase–polymerase chain reaction (RT-PCR) was performed on nasopharyngeal swabs at the Centers for Disease Control and Prevention of Guizhou Province. The researchers performed follow-up for all the patients, and the demographic data, clinical symptoms, and radiographic and laboratory results at admission were extracted from the electronic medical records.

*P* values were calculated with Pearson χ^2^ tests, Fisher exact tests, or Wilcoxon–Mann-Whitney *U* tests as appropriate; all tests were 2-sided. Statistical significance was set at *P* < .05. Data calculations were performed with SPSS statistical software version 22.0 (IBM Corp). Data analysis was performed in April 2020.

## Results

We examined data for 69 patients in total (median age, 33 years; range, 2-78 years; 35 male patients [50.7%]). Eleven of the patients (15.9%) had positive RT-PCR results for the COVID-19 nucleic acid test but without any symptoms. Among the 11 patients (median age, 27 years; range, 4-58 years), there were 7 male patients (63.6%), and 3 patients (27.3%) had comorbidities. Most of the 11 patients had moderate (9 patients) or mild infection (1 patient); only 1 patient was classified as having critical infection. The median interval from discharge to positive RT-PCR results was 14 days (range, 9-17 days). None of the patients were medical staff. There were no substantial differences in the demographic and baseline clinical characteristics between the recurrence group and nonrecurrence group (median age, 27 years [range, 4-58 years] vs 34 years [range, 2-78 years]; number of cluster cases, 8 patients [72.7%] vs 41 patients [70.7%]; presence of comorbidities, 3 patients [27.3%] vs 14 patients [24.1%]; median duration of hospital stay, 10 days [range, 7-24 days] vs 13 days [range, 7-38 days]) (Table 1). There also were no substantial differences between the recurrence group and nonrecurrence groups in terms of clinical symptoms (fever, 5 patients [45.5%] vs 26 patients [44.8%]; sore throat, 1 patient [9.1%] vs 4 patients [7.2%]; diarrhea, chill, anorexia, vomiting, and nausea, 0 patients vs 1 patient [1.7%] for all), radiographic findings (changes on chest computed tomography, 9 patients [81.8%] vs 36 patients [62.1%]), and laboratory values except for fatigue (4 patients [36.4%] vs 5 patients [8.6%]), number of initial symptoms (median, 2 symptoms [range, 0-4 symptoms] vs 1 symptom [range, 0-6 symptoms]), and creatine kinase level (median, 70.0 U/L [range 38.0-106.0 U/L] vs 46.0 U/L [range, 24.0-139.0 U/L]; to convert creatine kinase to microkatals per liter, multiply by 0.0167) (Table 2).

**Table 1.** Demographic and Baseline Clinical Characteristics of Patients With Coronavirus Disease 2019

Characteristic	Patients, No. (%)			χ^2^/*z*	*P* value
	Total (N = 69)	Recurrence (n = 11)	Nonrecurrence (n = 58)		
Male	35 (50.7)	7 (63.6)	28 (48.3)	0.873	.35^a^
Age, median (range), y	33 (2-78)	27 (4-58)	34 (2-78)	−0.730	.47^b^
Cluster cases	49 (71.0)	8 (72.7)	41 (70.7)	0.000	>.99^a^
Cluster cases, median (range), No.	4 (2-12)	5.5 (2-12)	3.5 (2-12)	−0.818	.41^b^
Underlying disease	17 (24.6)	3 (27.3)	14 (24.1)	0.000	>.99^a^
Clinical classification					
Severe-to-critical infection	8 (11.6)	1 (9.1)	7 (12.1)	0.000	>.99^a^
Mild-to-moderate infection	61 (88.4)	10 (90.9)	51 (87.9)		
Hospital stay, median (range), d	13 (7-38)	10 (7-24)	13 (7-38)	−0.897	.37^b^
Interval from discharge to positive reverse transcriptase–polymerase chain reaction reversal, median (range), d	NA	14 (9-17)	NA	NA	NA

Abbreviation: NA, not applicable.

^a^ Pearson χ^2^ test.

^b^ Wilcoxon–Mann-Whitney *U* test.

**Table 2.** Clinical Symptoms and Radiographic and Laboratory Findings for Patients With Coronavirus Disease 2019

Clinical symptoms and radiographic and laboratory findings	Patients, No. (%)			χ^2^/*z*	*P* value
	Total (N = 69)	Recurrence (n = 11)	Nonrecurrence (n = 58)		
Initial symptoms					
Fever	31 (44.9)	5 (45.5)	26 (44.8)	0.000	>.99^a^
Cough	30 (43.5)	8 (72.7)	22 (37.9)	3.250	.07^a^
Sputum production	17 (24.6)	5 (45.5)	12 (20.7)	1.866	.17^a^
Fatigue	9 (13.0)	4 (36.4)	5 (8.6)	4.067	.04^a^
Chest tightness	7 (10.1)	0	7 (12.1)	0.450	.50^a^
Sore throat	5 (7.2)	1 (9.1)	4 (7.2)	NA	>.99^b^
Nasal congestion	4 (5.8)	0	4 (6.9)	NA	>.99^b^
Runny nose	4 (5.8)	1 (9.1)	3 (5.2)	NA	.51 ^a^
Shortness of breath	4 (5.8)	0	6 (10.3)	NA	.58^b^
Headache	3 (4.3)	1 (9.1)	2 (3.4)	NA	.41^b^
Dizziness	3 (4.3)	2 (18.2)	1 (1.7)	NA	.06^b^
Sore muscles	2 (2.9)	1 (9.1)	1 (1.7)	NA	.30^b^
Diarrhea	1 (1.4)	0	1 (1.7)	NA	>.99^b^
Chill	1 (1.4)	0	1 (1.7)	NA	>.99^b^
Anorexia	1 (1.4)	0	1 (1.7)	NA	>.99^b^
Perspire while sleeping	1 (1.4)	0	1 (1.7)	NA	>.99^b^
Vomiting	1 (1.4)	0	1 (1.7)	NA	>.99^b^
Nausea	1 (1.4)	0	1 (1.7)	NA	>.99^b^
Initial symptoms, median (range), No.	1 (0-6)	2 (0-4)	1 (0-6)	−2.312	.02^a^
Changed chest computed tomography images	45 (65.2)	9 (81.8)	36 (62.1)	0.838	.36^a^
Laboratory values					
White blood cell count, median (range), ×10^9^/L	5.3 (2.8-12.1)	4.7 (3.2-7.7)	5.3 (2.8-12.1)	−0.792	.43^c^
Lymphocyte ratio, median (range), %	31.5 (12.3-65.7)	29.4 (25.2-65.7)	31.7 (12.3-65.7)	−0.091	.93^c^
Platelet count, median (range), ×10^9^/L	214.0 (96.0-486.0)	201.0 (97.0-327.0)	214.0 (96.0-486.0)	−0.282	.78^c^
Aspartate aminotransferase, median (range), U/L	18.0 (8.0-159.0)	20.0 (17.0-114.0)	18.0 (8.0-159.0)	−1.712	.09^c^
Alanine aminotransferase, median (range), U/L	23.0 (5.0-279.0)	33.0 (14.0-228.0)	23.0 (5.0-279.0)	−1.527	.13^c^
Creatine kinase, median (range), U/L	48.0 (24.0-139.0)	70.0 (38.0-106.0)	46.0 (24.0-139.0)	−2.183	.03^c^
Creatine kinase–MB fraction, median (range), U/L	10.0 (3.0-26.0)	12.0 (9.0-26.0)	10.0 (3.0-26.0)	−1.862	.06^c^
Serum creatinine, median (range), μmol/L	68.0 (25.0-99.1)	75.7 (28.2-85.7)	67.7 (25.0-99.1)	−0.541	.59^c^
C-reactive protein, median (range), mg/L	0.64 (0.02-25.41)	0.79 (0.09-12.70)	0.56 (0.02-25.41)	−1.049	.29^c^
Erythrocyte sedimentation rate, median (range), mm/h	17.0 (1.0-117.0)	14.5 (6.4-43.0)	17 (1.0-117.0)	−0.651	.52^c^

Abbreviation: NA, not applicable.

SI conversion factors: To convert alanine aminotransferase to μkat/L, multiply by 0.0167; aspartate aminotransferase to μkat/L, multiply by 0.0167; creatine kinase to μkat/L, multiply by 0.0167; creatine kinase–MB fraction to μg/L, multiply by 1.0.

^a^ Pearson χ^2^ test.

^b^ Fisher exact test.

^c^ Wilcoxon–Mann-Whitney *U* test.

## Discussion

On the basis of our follow-up results, 11 of 69 patients with COVID-19 showed positive RT-PCR results after discharge, which suggests that some recovered patients may still be virus carriers even after they reach the basic discharge criteria.^4^ Lan et al^5^ reported 4 patients with COVID-19, all medical staff in China, who presented with positive RT-PCR results 5 to 13 days after discharge. Although the participants in our research were not medical staff, our results revealed that the interval from discharge to positive RT-PCR results was 9 to 17 days (the intervals for 4 patients were >14 days), which is longer than the interval reported by Lan et al.^5^ Therefore, we suggest that medical institutions should pay attention to the follow-up of discharged patients by closely monitoring their RT-PCR results, even if they have been in quarantine for 14 days. In addition, our results revealed that fatigue, number of initial symptoms, and creatine kinase level could be associated with recurrent positive RT-PCR results, but further verification is required because of the limited number of patients.

This study was a single-center observational study limited to a small sample size, and 10 of the 11 patients had mild or moderate infection and only 1 patient was classified as having critical infection. Thus, these results may not be generalizable to other populations. Hence, it is necessary to conduct further studies to determine the factors associated with positive RT-PCR results after discharge.
